# Impact of Sunflower Press Cake and Its Modification with Liquid Glass on Polyurethane Foam Composites: Thermal Stability, Ignitability, and Fire Resistance

**DOI:** 10.3390/polym14214543

**Published:** 2022-10-26

**Authors:** Agnė Kairytė, Sylwia Członka, Jurga Šeputytė-Jucikė, Sigitas Vėjelis

**Affiliations:** 1 Laboratory of Thermal Insulating Materials and Acoustics, Faculty of Civil Engineering, Institute of Building Materials, Vilnius Gediminas Technical University, Linkmenu St. 28, LT-08217 Vilnius, Lithuania; 2 Institute of Polymer & Dye Technology, Lodz University of Technology, 90-924 Lodz, Poland

**Keywords:** bio-based polyurethane foam, sunflower press cake, liquid glass, flammability, ignitability, sustainability

## Abstract

Polyurethane (PUR) foams are some of the most promising thermal insulating materials because of their high flammability, but further applications are limited. Therefore, the development of flame-retardant materials with sufficient strength characteristics, water resistance, and low thermal insulating properties is of great importance to the modern building industry. This study evaluates the possibility of a vacuum-based liquid glass (LG) infusion into bio-based fillers, in this case, sunflower press cake (SFP) particles, to improve the mechanical performance, water absorption, thermal insulation, ignitability, thermal stability, and flame retardancy of the resulting polyurethane (PUR) foam composites. The main findings show that LG slightly improves the thermal stability and highly contributes to the ignitability and flame retardancy of the resulting products. Most importantly, from 10 wt.% to 30 wt.%, the SFP/LG filler reduces the thermal conductivity and water absorption values by up to 20% and 50%, respectively, and increases the compressive strength by up to 110%. The results obtained indicate that the proposed SFP/LG filler-modified PUR foam composites are suitable for applications as thermal insulation materials in building structures.

## 1. Introduction

Polyurethane (PUR) foams are an important group of polymers with many advantageous applications in various industries, such as for furniture, consumer bedding, automotive seats, cushioning, medicine, thermal insulation, sound absorption, and in specific locations where other materials would not be suitable because of the versatile chemistry of these products [1,2]. PUR is generally developed through the polyaddition reaction between isocyanates and polyols with other additives to improve the cellular structure and foaming [3]. The materials for the production of PURs are mainly from petrochemical feedstocks, whose usage is currently becoming unpopular because of the increasing environmental awareness and demand for a cleaner environment [4,5]. A global agreement has led to the use of raw materials from renewable resources or alternative routes to develop innovative green materials with improved or similar performance characteristics for various industries [6].

The PUR industry is no exception, and this has encouraged scientists and manufacturers globally to develop and produce composite foams from renewable feedstocks, more specifically isocyanates from fatty/amino acids [7], polyols from non-edible and edible natural oils [8,9] and natural fillers [10,11]. These natural or bio-based materials reduce greenhouse gas emissions and increase environmental conservation and sustainability, and importantly, are a cost-efficient way to develop and produce PUR composites. Natural fillers, currently, have gained considerable worldwide attention due to their ability to improve the performance characteristics of the final products. Several studies suggest that the incorporation of natural fillers such as primrose oil cake and walnut shells provide PUR foam composites with improved mechanical performance, thermal stability, and thermal insulation [12,13].

However, biobased PUR foams and PUR foam composites are easily ignitable, burn fast, and release smoke, heat, carbon dioxide, and carbon monoxide during combustion. To protect the environment, human life, and property, it is important to endow such PUR foams and composites with flame retardancy and smoke suppression. There have been many studies focusing on the enhancement of the flame retardancy and reduction in smoke release, and the most frequently mentioned are montmorillonite [14], expandable graphite [15], carbon nanotubes [16], carbon microspheres [17], etc. Additionally, naturally occurring modified structures, e.g., lignin [18], phytic acid [19], chitosan [20], and starch [21], for the synthesis of flame retardants, have also proved to be suitable for implementation due to several hydroxyl groups. Another group of potential flame retardants are the glass-based fillers and modifiers such as hollow glass microspheres [22]. These have shown excellent flame retardant, smoke suppression, and thermal insulation properties, indicating the feasibility of using glass and glass-based materials for a specific application. Additionally, a previous study [23] decided to use a simpler and more widely available material, namely, liquid glass (LG), which was incorporated on the surface of PUR foams to reduce the flammability of the resultant products.

Therefore, the current study analyses the possibility of using LG as a coating material for bio-based fillers—sunflower press cake (SFP) particles—to increase the flame retardancy, ignitability, and thermal stability without deteriorating the other performance characteristics of PUR foam composites. Here, 10–30 wt.% SFP particles were vacuum-infused with LG and incorporated into PUR foam composites. Additionally, all the PUR foam composites were compared with commercially available PUR foams with a tris chloroisopropyl phosphate (TCPP) flame retardant in order to evaluate the effectiveness of the LG. Moreover, the current study was a continuation of a study conducted by Kairytė et al. [24], where the results of the physical and mechanical properties of SFP/LG filler-modified foams without a flame retardant were presented and discussed.

## 2. Materials and Methods

### 2.1. Materials

The following two polyols were incorporated to produce a control PUR foam and PUR foam composites: the rapeseed oil-based polyol BioPolyol RD (SIA PolyLabs, Riga, Latvia) with OH_v_ = 350 KOH/g and the sucrose-based polyol PETOL 400-4G (Oltchim, Râmnicu Vâlcea, Romania) with OH_v_ = 421 KOH/g. Polymeric 4,4-diphenylmethane diisocyanate (pMDI) Lupranat M20S with an NCO content of 31.5% was purchased from BASF, Ludwigshafen, Germany. Distilled water was used as an eco-friendly blowing agent. Polycat 9 (Air Products and Chemicals, Inc., Allentown, PA, USA) was used to catalyse the reactions during foaming. A silicone surfactant ST-52 (Shijiazhuang Chuanghong Technology Co., Ltd., Shijiazhuang, China) was incorporated to obtain a regular cellular structure. Tris chloroisopropyl phosphate TCPP (Lanxess, Germany) was used as a flame retardant. SFP was used as a PUR foam filler, and it was purchased from a local company in Vilnius, Lithuania. Before the treatment with LG, it had the moisture content of 1.1 wt.%, a particle size from 0.063 mm to 1.4 mm and a bulk density of 527 kg/m^3^. After treatment with the LG, it had a moisture content of 0.50 wt.%, a particle size from 0.09 mm to 2.8 mm and a bulk density of 531 kg/m^3^. The LG (JSC Lerochemas, Klaipeda, Lithuania) had the mass fraction of Na_2_O—29% and SiO_2_—29%.

### 2.2. Preparation of the Control PUR Foams and PUR Foam Composites

At first, the SFP was prepared before its use in the PUR foams. It was crushed, milled and dried at 110 °C for 24 h to remove the excess moisture. The dried SFP particles were then further mixed with LG at a ratio of 1:1 and vacuum-impregnated. The impregnation procedure was carried out in five cycles with holding at 1 bar for 10 min. Furthermore, the obtained viscous mass was dried at 105 °C for 24 h.

Firstly, component A (i.e., the polyols, catalyst, surfactant, and distilled water) was divided into six parts. The first part was used to prepare the control PUR foams (SFP-0), the second part was used for the PUR foams with TCPP (SFP-0/TCPP), and the third part was used for the PUR foam composites with 10 wt.%, 20 wt.%, and 30 wt.% non-impregnated SFP particles (SFP-10, SFP-20, and SFP-30, respectively). The fourth part was used for the PUR foam composites with 10 wt.%, 20 wt.%, and 30 wt.% non-impregnated SFP particles and TCPP (SFP-10/TCPP, SFP-20/TCPP, and SFP-30/TCPP, respectively), the fifth part was used for the PUR foam composites with 10 wt.%, 20 wt.%, and 30 wt.% LG-impregnated SFP particles (SFP-10/LG, SFP-20/LG, and SFP-30/LG, respectively), and the sixth part was used for the PUR foam composites with 10 wt.%, 20 wt.%, and 30 wt.% LG-impregnated SFP particles and TCPP (SFP-10/LG/TCPP, SFP-20/LG/TCPP, and SFP-30/LG/TCPP, respectively). The compositions of each PUR foam composite are presented in Table 1.

The prepared six parts from component A were thoroughly mixed with isocyanate (index 125) for 10 s at 1800 rpm. The prepared final mixtures were immediately poured into the open moulds and left to cure at (23 ± 2) °C and (50 ± 5)% relative air humidity conditions.

### 2.3. Testing Methods

#### 2.3.1. Microstructural Analysis

For the microstructural examination of the SFP/LG filler, the control PUR, flame retardant PUR foam, non-flame retardant/flame retardant PUR foam composites, and char residues, a scanning electron microscope (SEM) JEOL SM–7600F (JEOL Ltd., Tokyo, Japan) was implemented. The samples were prepared with a thin gold layer in order to further proceed with the imaging.

The percentage volume of the closed cells was determined based on ISO 4590 [25], method 2 requirements for three 100 mm × 30 mm × 30 mm-sized samples. Before the measurements, all the samples were kept for conditioning for 16 h at (23 ± 2) °C and (50 ± 5)% relative air humidity conditions. Measurements for each composition were carried out at (23 ± 2) °C and (50 ± 5)% relative air humidity conditions.

#### 2.3.2. Mechanical Performance

The apparent density of the samples was determined in accordance with EN 1602 [26] requirements.

The compressive strength test was conducted according to the requirements of EN 826 [27] using a universal testing machine H10KS Hounsfield (Tinius Olsen Ltd., Surrey, UK). The test was conducted on three samples with a size of 50 mm × 50 mm × 50 mm for each composition. According to the requirements, the samples were conditioned for 6 h and the test was carried out at (23 ± 5) °C.

A dynamic mechanical analysis was carried out in an ARES rheometer (TA Instruments, New Castle, DE, USA). The temperature range used for the measurements was in the range of 40–250 °C, with a heating rate of 10 °C/min, frequency of 1 Hz and the constant strain of 0.1%.

#### 2.3.3. Water Resistance Properties

The short-term water absorption was determined based on ISO 29767 [28], method B methodology. For the test, four 200 mm × 200 mm-sized samples for each composition were used. Before the test, all the samples were kept for conditioning for not less than 6 h at (23 ± 5) °C. A partial immersion in water was carried out for 24 h.

#### 2.3.4. Thermal Properties

The thermal conductivity was tested for three 300 mm × 300 mm × 50 mm-sized samples of each composition according to the methodology indicated in EN 12667 [29] using a heat flow meter FOX 304 (TA Instruments, Eden Prairie, MN, USA). The heat flow direction during the test was upwards. The measurements were taken at an average temperature of 10 °C, and the temperature difference between the plates was 20 °C. The samples from all compositions were conditioned at (23 ± 3) °C and (50 ± 10)% relative air humidity conditions for not less than 16 h, as indicated in the harmonised product standard EN 14315-1, Annex C.

The thermal properties of the control PUR foams and PUR foam composites were determined by a thermogravimetric analysis (TGA) and differential thermogravimetric analysis (DTG) using an apparatus STA 449 F1 Jupiter Analyser (Netzsch Group, Selb, Germany). The samples were tested at 10 °C/min speed in an argon atmosphere in the 25–600 °C temperature range. The decomposition temperatures at 5% and 50% weight loss (T_5%_ and T_50%_), as well as the peak temperatures, were determined.

#### 2.3.5. Fire Retardance Properties

The reaction to fire of the control PUR foams and PUR foam composites was conducted according to ISO 5660-1 [30] with a cone calorimeter Cone 2a (Atlas Electric Devices Co., Chicago, IL, USA). The parameters used for the test were as follows: a heat flow of 35 kW/m^2^, surface area of 88.4 cm^2^, and overall testing time of 1200 s. As a result, the heat release rate (HHR), total smoke production per unit area (TSR), carbon monoxide yield (COY), and carbon dioxide yield (CO_2_Y) were reported and the average values were calculated.

The ignitability test was performed on three 200 mm × 100 mm × 50 mm-sized samples from each composition according to ISO 11925-2 [31] requirements. All the samples were treated with an open flame directed at an angle of 45° for 15 s. When the flame source was removed, the samples were additionally left to flame for 5 s and the corresponding ignitability parameters were determined.

For the limited oxygen index (LOI) determination, an oxygen index instrument (“NETZSCH TAURUS Co.”, Ltd., Weimar, Germany) was used. A tip of the sample was ignited for 5 s with a gas burner having a propane–butane mixture. The LOI was calculated as the percentage of oxygen and nitrogen volume in the mixture.

## 3. Results and Discussion

### 3.1. The Main Physical Properties of the Control PUR Foams and PUR Foam Composites

A low density, water absorption and thermal conductivity and a high mechanical performance are very desirable properties for building thermal insulation. Table 2 presents the physical properties and structural parameters of the PUR and PUR foam composites. The performance characteristics in Table 2 of the SFP/LG-modified PUR foam composites without TCPP as a flame retardant were previously presented and thoroughly discussed in a study by Kairytė et al. [24]. They were selected and shown in the current study to better describe the difference between PUR foam composites with and without a flame retardant.

As expected, the SFP and SFP/LG filler, regardless of the amount added, increased the apparent density of the resulting PUR foam composites with and without TCPP compared to the control PUR foam (SFP-0 and SFP-0/TCPP). Such an increase for the PUR foam composites was also confirmed by numerous studies [32,33,34]; however, an interesting observation was made regarding the LG-modified SFP filler. Compared to the PUR foam composites with an SFP filler (SFP and SFP/TCPP), the SFP/LG and SFP/LG/TCPP composites had a lower apparent density. According to the literature [24,35], the main factor might be the increasing initial dynamic viscosity of the mixtures with unmodified SFP fillers.

Moreover, changes in the apparent density might have influenced the mechanical performance of the final products. Table 2 presents the average values of the compressive strength of the control PUR and non-flame retardant and flame retardant PUR foam composites with SFP and SFP/LG. It can be clearly seen that the incorporation of SFP and SFP/LG fillers into the PUR matrix led to the improvement in the compressive strength values. The SFP filler increased the strength characteristic by a maximum of 99% for the formulations without the TCPP and by 88% for the formulations with the TCPP, while the SFP/LG filler increased the strength characteristics by a maximum of 91% for the formulations without the TCPP and by a maximum of 43% for the formulations with the TCPP. Paciorek-Sadowska et al. [36] studied the impact of rapeseed press cake on the performance characteristics of PUR foams and noted that the compressive strength was impacted not only by the apparent density but also by the addition of the bio-filler and their homogeneous distribution throughout the structure, which contributed to the reinforcement of the walls and struts of the PUR matrix.

It can also be observed from Table 2 that the SFP and SFP/LG fillers positively affected the water absorption of the non-flame retardant and flame retardant PUR foam composites. The parameter reduced by up to 50% for all compositions, indicating sufficient water barrier properties of the products. This can be explained by the fact that the SFP filler had a residual oil of up to 6 wt.% [37]; therefore, this residual oil repelled water molecules from penetration into the filler particles and impeded the increase in the short-term water absorption of the resultant products.

Thermal conductivity is a parameter that characterizes thermal insulating materials, and its value can be impacted by numerous factors such as the apparent density, closed cell content, average cell size, thickness of the insulating layer tested, blowing agent, and the nature of the fillers.

**Figure 1:**
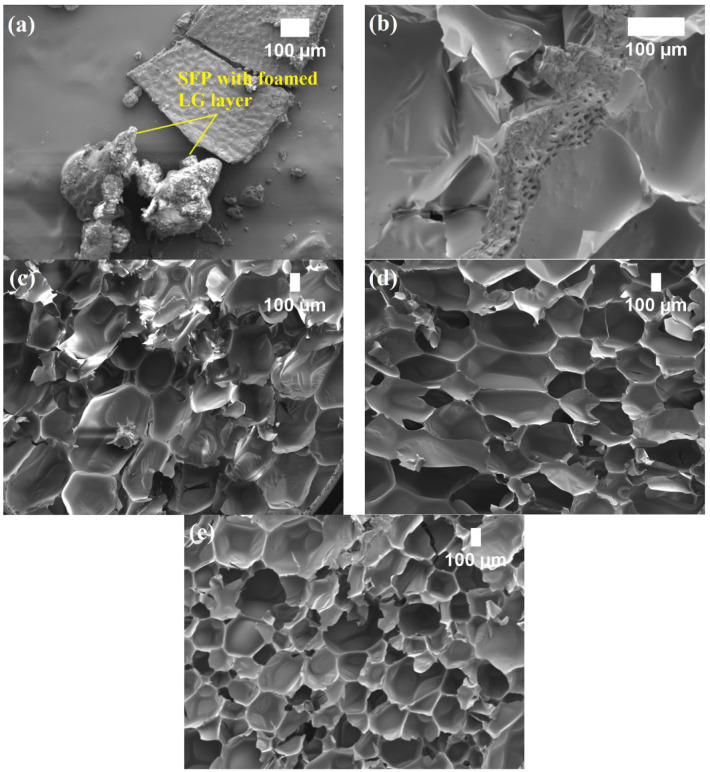
LG-modified SFP filler in PUR matrix: (**a**) LG-modified SFP filler (magnification × 100); (**b**) LG-modified SFP filler in PUR foam matrix (magnification × 200); (**c**) SFP-10/LG sample matrix (magnification × 40); (**d**) SFP-20/LG sample matrix (magnification × 40); and (**e**) SFP-30/LG sample matrix (magnification × 40).

SEM images of the flame retardant PUR foams revealed that the SFP and SFP/LG fillers (Figure 1a) were located in the cell struts (Figure 1b), which changed the morphology of the porous structure. Compared to the control PUR foam, the incorporation of the SFP filler or LG-modified SFP filler into the PUR matrix slightly reduced the thermal conductivity value due to the ability of the filler to act as a nucleation agent. The studies on the effect of specific fillers such as cellulose [38] on the cell structure of PUR foam composites show that the introduction of the filler into a PUR system reduced the size of the cells (Figure 1c,e). These results were found to be in agreement with the studies carried out by Leszczyńska et al. [39], who demonstrated a reduction in cell size after the incorporation of vegetable-based fillers. The smaller cell size could have effectively extended the heat transfer path through the solid phase and reduced the radiant heat transfer, thus, lowering the thermal conductivity values of the non-flame retardant and flame retardant PUR foam composites with the SFP and SFP/LG fillers. Additionally, the percentage of the closed cell content had a significant effect on the thermal conductivity value. Comparing the SFP-0, SFP-30, and SFP-30/LG foams, the parameter increased from 81 vol.% to 85 vol.% and 92 vol.%, respectively. Such changes in the cellular structure highly influenced the reduction in the thermal conductivity values, i.e., from 0.0354 W/(m·K) to 0.0321 W/(m·K) and 0.0319 W/(m·K), respectively. Similar tendencies could be observed for the formulations with the TCPP.

**Figure 2:**
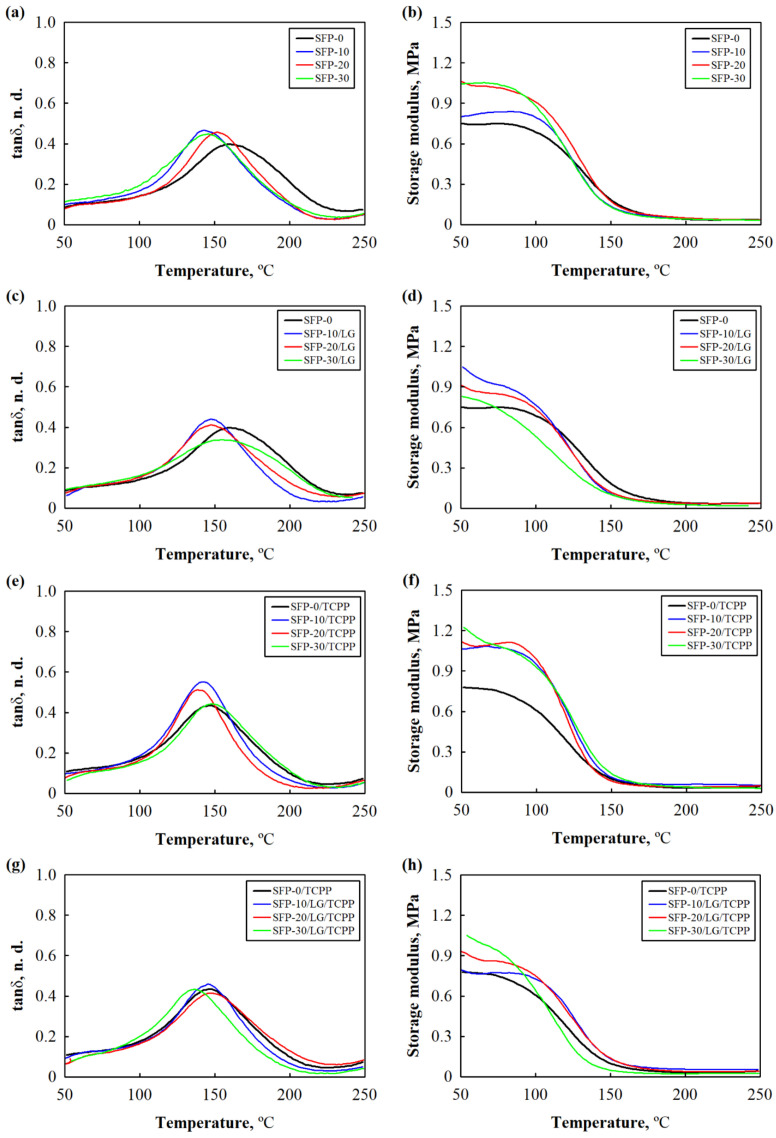
Dynamic mechanical analysis curves of PUR foam composites: (**a**,**c**,**e**,**g**) tanδ and (**b**,**d**,**f**,**h**) storage modulus.

Figure 2 presents the temperature plots of the tanδ and storage modulus for the non-flame retardant and flame retardant PUR foam composites with SFP and LG/SFP fillers obtained during the dynamic mechanical analysis. According to the results presented in Figure 2, the incorporation of the LG-modified or unmodified SFP filler into the PUR foam composites affected the value of T_g_. Compared to SFP-0, after the addition of 10–30 wt.% of filler, the value of T_g_ shifted towards lower temperatures. The highest value of T_g_ was still observed for the control PUR foam composite (SFP-0), which was 160 °C. A 30 wt.% of the SFP (Figure 2a) and LG/SFP (Figure 2c) fillers decreased the temperature to 152 °C and 154 °C, respectively. This effect may be associated with the insufficient interphase bonding between the surface of the SFP and LG/SFP fillers and the PUR foam matrix, which contributed to an increase in polymer chains’ mobility. Similar results were shown in the study by Członka et al. [40] for PUR foam composites reinforced with a casein/apricot filler; however, when comparing the PUR foam composites with the SFP and LG/SFP fillers, the improvement in the T_g_ value could be observed and the effectiveness of an SFP filler-modification with LG was confirmed.

Slightly different observations could be found for the flame retardant PUR foam composites with SFP and LG/SFP fillers (Figure 2e,g). Compared to the control flame retardant PUR foam (SFP-0/TCPP), a 30 wt.% SFP filler increased the T_g_ from 145 °C to 149 °C, while a 20 wt.% LG/SFP filler increased the temperature to 147 °C.

The improved performance can be explained by the fact that the addition of TCPP reduced the dynamic viscosity of the mixture, thus, resulting in the formation of a more uniform microstructure of the flame retardant PUR foam composites.

According to the results presented in Figure 2b,d,f,h, the incorporation of SFP and LG/SFP fillers into non-flame retardant and flame retardant PUR foam composites increased the value of the storage modulus. Apparently, additional hydroxyl groups present on the surface of an SFP filler and cause more rigid composites, which express an increase in the storage modulus values. For example, the addition of 10–30 wt.% SFP and LG/SFP fillers increased the parameter by up to 41% in the non-flame retardant PUR foam composites and by up to 54% in the flame retardant PUR foam composites. According to Olszewski et al. [41], the stiffening of the composites can be explained by the additional cross-linking caused by a higher amount of isocyanate used in the adjusted formulations.

### 3.2. Thermal Stability of the Control PUR Foams and PUR Foam Composites

**Figure 3:**
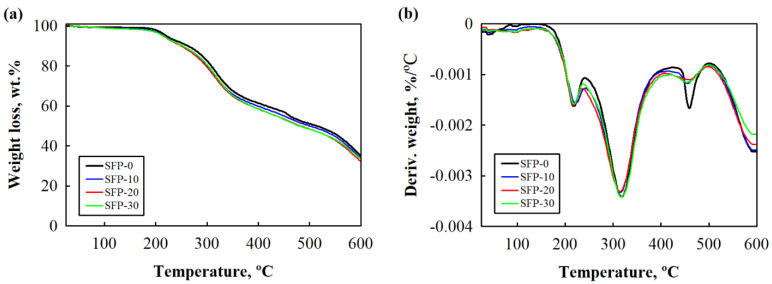

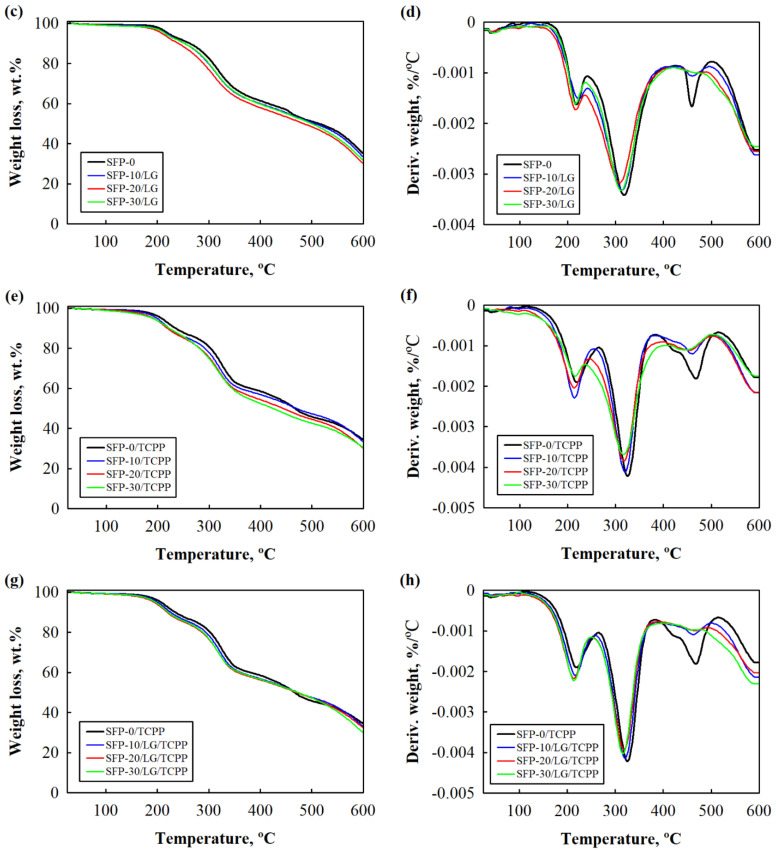
Thermal stability of the control PUR and flame retardant PUR foams: (**a**,**b**) TGA and DTG curves of PUR foams with SFP filler; (**c**,**d**) TGA and DTG curves of PUR foams with LG-modified SFP filler; (**e**,**f**) TGA and DTG curves of flame retardant PUR foams with SFP filler; and (**g**,**h**) TGA and DTG curves of flame retardant PUR foams with LG-modified SFP filler.

The thermal degradation of PUR and its composites is presented in the TGA/DTG curves in Figure 3 and the main parameters in Table 3. Decomposition was observed to take place in three stages. The first stage involved the degradation of urethane bonds at approximately 150–220 °C (the total weight loss was around 20%). Furthermore, the substantial weight loss due to the decomposition of soft polyol segments and the SFP as well as the SFP/LG fillers was observed at a temperature range of 220–400 °C [2,42]. In this temperature range, the percentage weight loss was found to be up to 50% [43]. Above 400 °C, previously generated fragments began to degrade with a loss of weight of nearly 70% until 600 °C. The results were as expected—the SFP and SFP/LG filler-modified composites displayed similar thermal degradation trends compared to the control PUR foam.

However, Table 3 shows that at temperatures at 5% and 50% weight loss (T_5wt.%_ and T_50wt.%_), the non-flame retardant and flame retardant SFP/LG filler-modified PUR foams had a slightly improved thermal stability compared to the PUR foams with only an SFP filler. This might be due to the solid SFP/LG filler particles incorporated in the cellular foam structure, which reduced the heat transfer and slightly slowed down the thermal degradation of the PUR foam composites [44].

Contrary to the results presented in the study by Bryśkiewicz et al. [45], who analysed the impact of walnut and hazelnut shells on the thermal stability of flexible PUR foams, significant improvements in the thermal stability of the non-flame retardant and flame retardant SFP and SFP/LG-modified PUR foam composites were not detected, which could also be confirmed by the results of the char yield at 600 °C. Compared to the control PUR foam, all the PUR foam composites had an insignificantly reduced char yield with an increase in the amount of SFP and SFP/LG fillers.

This was related to the cellulose and hemicellulose in the SFP and SFP/LG fillers [46]. These polysaccharides expedite the conversion of volatile gases from PUR. Slightly lower values for T_max1_ were suspected for the PUR foam composites with SFP and SFP/LG fillers and were related to the insufficient dispersion and formation of clusters into the PUR system, which can alter the cross-link density of the products [47]. However, the addition of the non-flame retardant SFP or SFP/LG filler did not change the temperature in the first stage, indicating a sufficient filler dispersion and cross-link density of the resultant PUR foam composites, while the incorporation of flame retardant fillers gave rather lower results. The T_max2_ was observed to be lower for the SFP and SFP/LG filler-modified PUR foam composites. This could be associated with the fact that at this stage, cellulose (~25 wt.% SFP), hemicellulose (~15 wt.% SFP), and lignin (~9 wt.% SFP) start to degrade [48]. However, the degradation peak temperature associated with the third stage indicated that the LG slightly shifted towards the DTG curve compared to the control PUR foam (SFP-0), which could be related to the fact that the reaction between the isocyanate and the SFP/LG filler cross-linked the polymer structure of the non-flame retardant and flame retardant PUR foam composites.

Furthermore, the degradation rate of the non-flame retardant and flame retardant SFP/LG filler-modified PUR foam composites in the second stage (Figure 3d,h) was lower compared to the control PUR foam and PUR foam composites with the unmodified SFP filler. This effect can be explained by the assumption made by several authors [49] that fillers such as SFP/LG are able to absorb part of the heat generated during thermal degradation.

### 3.3. Ignitability and Flammability of the PUR and Flame Retardant PUR Foams

**Figure 4:**
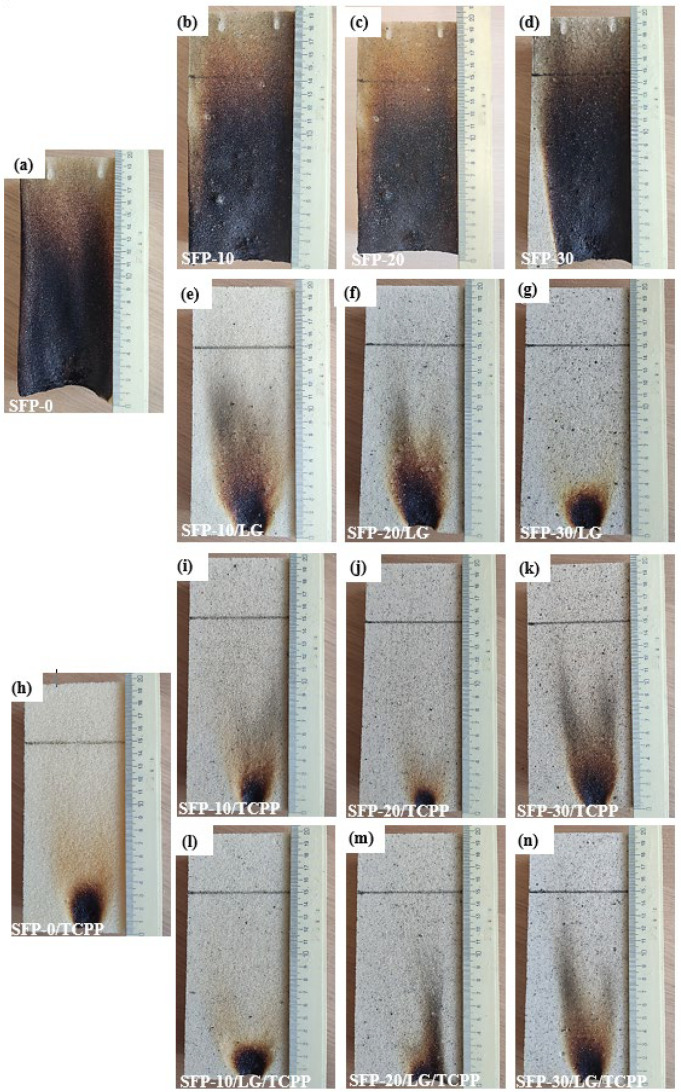
Ignitability experiment on: (**a**) SFP-0; (**b**) SFP-10; (**c**) SFP-20; (**d**) SFP-30; (**e**) SFP-10/LG; (**f**) SFP-20/LG; (**g**) SFP-30/LG; (**h**) SFP-0/TCPP; (**i**) SFP-10/TCPP; (**j**) SFP-20/TCPP; (**k**) SFP-30/TCPP; (**l**) SFP-10/LG/TCPP; (**m**) SFP-20/LG/TCPP; and (**n**) SFP-30/LG/TCPP.

Small-scale ignitability tests were implemented; therefore, Figure 4 presents images of the samples after the ignitability test under an open flame, while Table 4 presents the test parameters. As more SFP particles were recycled in the PUR foam composites, a faster ignitability was observed.

The height of the flame damaged area increased, and self-extinguishment was not achieved after removal of the flame source; therefore, the samples failed to pass the test. This was not a surprise because SFP is prone to heating and spontaneous ignition. A remarkable improvement was observed for the SFP/LG filler-modified PUR foam composites. Even at a 10 wt.% of SFP/LG filler, the height of the damaged area reduced from 180 mm to 46 mm and to 29 mm when 30 wt.% of the SFP/LG filler was incorporated into the PUR matrix. Instant self-extinguishment was also observed for the SFP/LG filler-modified PUR foam composites, which was also characteristic to the PUR foams with a TCPP flame retardant. This effect might be explained by the ability of the thin-foamed LG layer on the SFP particle (Figure 1a) to act as a flame barrier [50] which inhibited the flame spread and high temperature and which led to growth in the more thermally-stable soft segments of the PUR foam composites [51].

In such a case, the 30 wt.% SFP/LG filler gave almost the same results as the 30 wt.% uncoated SFP filler or a control PUR foam with a TCPP flame retardant (SFP-30/TCPP and SFP-0/TCPP). Moreover, Table 4 indicates that even better results were obtained for the flame retardant SFP/LG filler-modified PUR foam composites (SFP-10/LG/TCPP—SFP-30/LG/TCPP) compared to the flame retardant PUR foam composites without LG (SFP-10/TCPP—SFP-30/TCPP). The obtained results allow us to state that the LG and TCPP flame retardant worked better together in the PUR foam composites rather than separately, thus, indicating a synergy effect after ignition and during flame spread.

**Figure 5:**
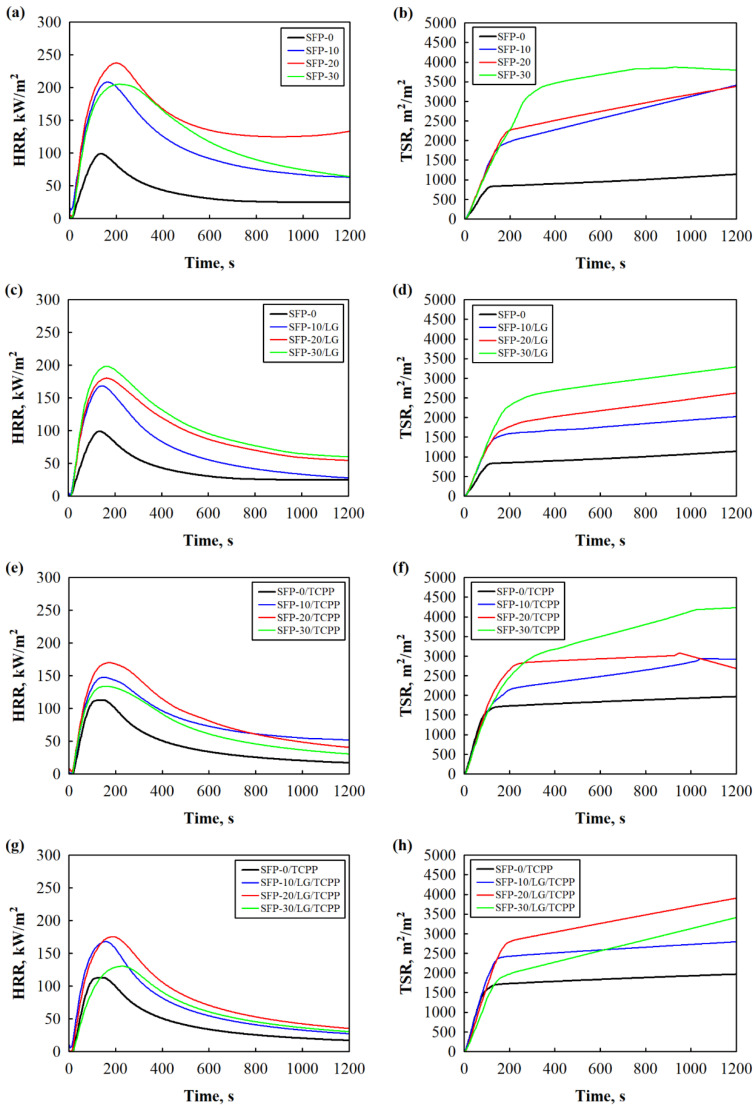
HRR and TSR of: (**a**,**b**) PUR foams with SFP filler; (**c**,**d**) PUR foams with LG-modified SFP filler; (**e**,**f**) flame retardant PUR foams with SFP filler; and (**g**,**h**) flame retardant PUR foams with LG-modified SFP filler.

The cone calorimeter test is considered one of the best technical measures to evaluate the combustion behaviour and to obtain related characteristics of all kinds of materials for fire-related properties [52,53,54].Consequently, the control PUR foam and all PUR foam composites were tested, and their HRR and TSR curves are presented in Figure 5 while the main parameters are shown in Table 4.

Compared to the control PUR foam (SFP-0), it is possible to observe from Table 5 that the ignition time showed a decrease from 4 s to 2 s when the unmodified SFP filler was used. Similar results were observed by Miedzińska et al. [55] in PUR foam composites with a vermiculite filler coated with casein, chitosan, and potato protein. As mentioned before, this behaviour may be explained by a large amount of flammable matter in the fillers or their coatings; however, based on the results of the SFP filler, some improvements may be observed for the SFP/LG filler regardless of the amount incorporated in the PUR foam composites.

For a short period of time, the LG on the SFP particles developed a thin barrier, thus, slightly increasing the ignition time; however, compared to the non-flame retardant PUR foam (SFP-0) and flame retardant PUR foam (SFP-0/TCPP), no visible improvements were observed for the SFP and SFP/LG fillers-modified PUR foam composites.

All the non-flame retardant and flame retardant PUR foam composites showed higher values for HRR compared to the control PUR foams (SFP-0 and SFP-0/TCPP), which may be related to the higher flammability of the composites and decomposition of LG and SFP. Apart from the control PUR foams, the SFP/LG filler showed the ability to reduce the average HRR values by up to approximately 25% compared to the SFP filler. Similar tendencies could be observed for the TSR results as well as for both the non-flame retardant and flame retardant PUR foam composites.

This can be explained by the fact that the LG can impart fire retardant properties because it forms foam-like crystals on the surface of SFP and provides some kind of insulating barrier between the filler particle and the flame [56].

When the results for the total amount of COY and CO_2_Y were evaluated, higher values were observed for the SFP and SFP/LG filler-modified PUR foam composites compared to the control one. The ratio increased from 0.05 to a maximum of 0.06 for the SFP filler, to 0.09 for the SFP filler with TCPP, and to 0.08 for the SFP/LG filler with TCPP. Generally, as the COY/CO_2_Y ratio value becomes higher, the combustion of PUR foam composites is more incomplete and a greater amount of toxic smoke is emitted. All the non-flame retardant and flame retardant PUR foam composites showed higher values for the COY/CO_2_Y ratio, except for the ones with the SFP/LG filler, which was 40% lower compared to the control PUR foam. In addition, the PUR foam composites presented lower HRR and COY/CO_2_Y ratio values compared with other studies related to the modification of PUR foam fillers [57,58]. Additionally, the results for the LOI are also presented in Table 5. Obviously, the control PUR foam (SFP-0) was a flammable material, and its LOI value was only 19.8%. The results of the SFP filler-modified PUR foam composites show that the addition of 10–30 wt.% of SFP reduced the LOI value to 18.9%, which is in a great agreement with the study carried out by Borowicz et al. [59], and Członka et al. [47]. Interestingly, the LOI values of the non-flame retardant PUR foam composites with LG/SFP and flame retardant PUR foam composites with only SFP were the same irrespective of the filler amount added. These results show that LG gives almost the same effect as TCPP. Very interesting results could be seen for the flame retardant PUR foam composites with the LG/SFP filler, where even 10 wt.% of LG/SFP filler increased the LOI value to 21.7% indicating that LG and TCPP work together better than separately.

### 3.4. Analysis of Char Residues

**Figure 6:**
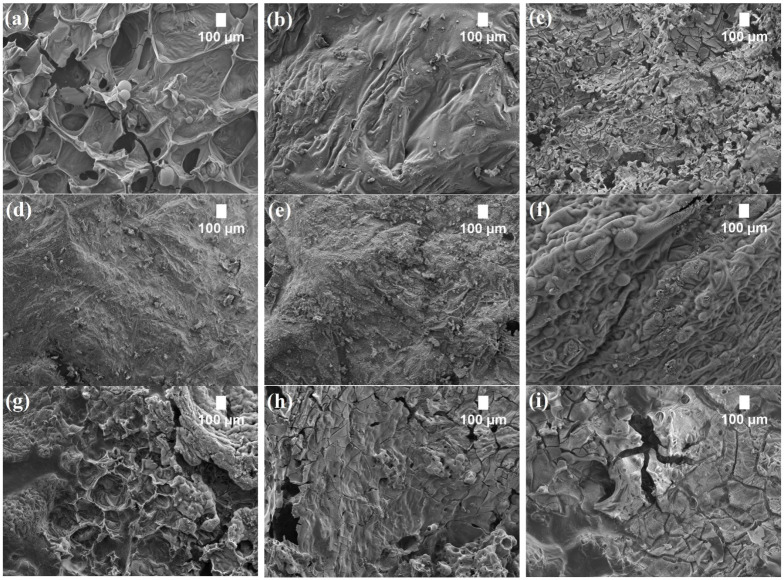

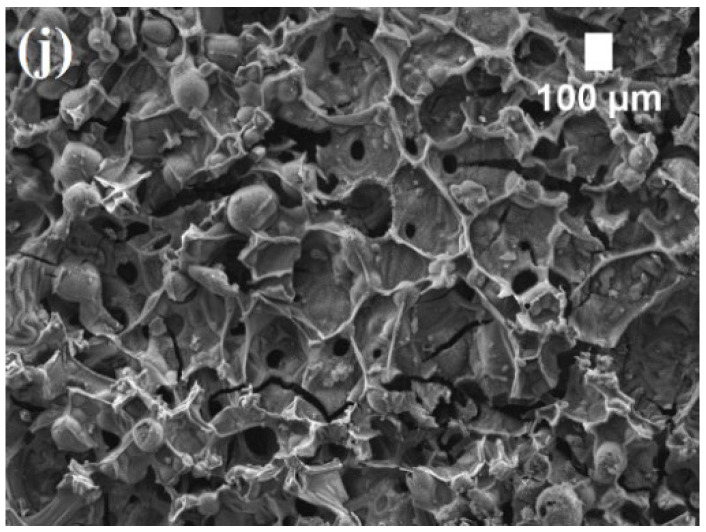
SEM images of char residues after the cone calorimeter test (magnification × 50): (**a**) SFP-0; (**b**) SFP-10; (**c**) SFP-30; (**d**) SFP-10/LG; (**e**) SFP-30/LG; (**f**) SFP-0/TCPP; (**g**) SFP-10/TCPP; (**h**) SFP-30/TCPP; (**i**) SFP-10/LG/TCPP; and (**j**) SFP-30/LG/TCPP.

In order to understand how the structure of char after cone calorimetry tests determine some flame suppression parameters, scanning electron microscopy of the resulting char residues was implemented and the images are shown in Figure 6. The control PUR foam without TCPP (Figure 6a) and the PUR foam composites with 10 wt.% and 30 wt.% of SFP filler (Figure 6b,c, respectively) displayed a foamed char structure with smaller and larger voids, indicating an ineffective barrier layer. The fire resistance of such foams and composites decreases due to the generated heat and fire, which penetrate the deeper layers of a structure. Consequently, such a structure of char residues indicates that such char cannot efficiently retard the mass and heat transfer during combustion [60,61].

Contrary results were obtained for the PUR foam composites with an SFP/LG filler (Figure 6d,e). The modification of SFP particles with LG allows for the formation of a continuous, smooth, and intact char layer without holes or even small cracks. Such a char layer forms a barrier and prevents oxygen from reaching the underlying composite layers [62]. A very similar view could be observed for the control PUR foam with TCPP (Figure 6f). This is in great agreement with the study by Yuan et al. [63], where they concluded that the phosphoric acids in TCPP promote the formation of a more compact carbonization zone by dehydration; however, the destruction of the char layer was observed for the flame retardant PUR foam composites with the SFP filler (Figure 6g,h). No continuous or intact zones were formed, and many voids and cracks were visible. This can be attributed to the flammable matter in the SFP filler. Furthermore, the char layer of the flame retardant PUR foam composites with 10 wt.% SFP/LG filler (Figure 6i,j) showed quite a dense structure with visible cracks, while the char structure of the 30 wt.% SFP/LG filler was characterized by denser and smaller cells without cracks and visible voids.

## 4. Conclusions

Non-flame retardant and flame retardant PUR foams were modified with 10–30 wt.% sunflower press cake filler particles after a vacuum-based impregnation with liquid glass. The effect of such a filler on the fire resistance properties of PUR foam composites, such as the performance characteristics (i.e., the apparent density, thermal conductivity, short-term water absorption, and compressive strength), thermal stability, and fire resistance (i.e., ignitability and cone calorimetry parameters) were investigated.

The results show that liquid glass-coated sunflower press cake filler particles increase the apparent density and the compressive strength by up to 87% and 110%, respectively, and reduce the thermal conductivity and short-term water absorption values by up to 18% and 50%, respectively, for non-flame retardant and flame retardant PUR foam composites.

Ignitability, cone calorimetry tests, and char layer analysis show that LG leads to a reduced negative impact of the SFP filler on the flame retardant properties of PUR foam composites. The combination of an SFP/LG filler and TCPP could provide some synergy effect, which is based on the ability of the SFP/LG filler to form a denser cellular structure and on TCPP to cause the formation of a consistent char layer, thus, increasing the ignition time by up to 8 s and reducing the height of the damaged area by up to 19 mm, the heat release rate by up to 37%, and the total smoke released by 24%.

The obtained results for the PUR foams with sunflower press cake filler particles, show that it is worth using liquid glass as an effective option for a coating to obtain a building material that could be promising for future applications in building envelopes as a thermal insulating layer.

## Figures and Tables

**Table 1:** Compositions of PUR foam composites with and without flame retardant.

Foam	Raw Materials, Parts by Weight							
	BioPolyol RD	PETOL 400-4G	Lupranat M20S	Distilled Water	Polycat 9	ST-52	TCPP	SFP, wt.% SFP/LG, wt.%
Control PUR foams								
SFP-0	60	40	Index 125	2.7	1.0	3.0	–	–
SFP-0/TCPP							25	–
PUR foam composites with SFP filler								
SFP-10	60	40	Index 125	2.7	1.0	3.0	–	10
SFP-20								20
SFP-30								30
PUR foam composites with LG-modified SFP filler								
SFP-10/LG	60	40	Index 125	2.7	1.0	3.0	–	10
SFP-20/LG								20
SFP-30/LG								30
PUR foam composites with TCPP and SFP filler								
SFP-10/TCPP	60	40	Index 125	2.7	1.0	3.0	25	10
SFP-20/TCPP								20
SFP-30/TCPP								30
PUR foam composites with TCPP and LG-modified SFP filler								
SFP-10/LG/TCPP	60	40	Index 125	2.7	1.0	3.0	25	10
SFP-20/LG/TCPP								20
SFP-30/LG/TCPP								30

**Table 2:** Physical properties of control PUR foams and PUR foam composites.

Foam	Parameter				
	Apparent Density, kg/m^3^	Closed Cell Content, vol. %	Thermal Conductivity, W/(m·K)	Compressive Strength, kPa	Short-Term Water Absorption, kg/m^2^
Control PUR foams					
SFP-0	39 ± 3	81 ± 2	0.0354 ± 0.0003	168 ± 5	0.36 ± 0.02
SFP-0/TCPP	41 ± 4	82 ± 3	0.0356 ± 0.0003	246 ± 7	0.18 ± 0.03
PUR foam composites with SFP filler					
SFP-10	41 ± 4	87 ± 2	0.0322 ± 0.0004	224 ± 5	0.17 ± 0.02
SFP-20	66 ± 5	90 ± 1	0.0294 ± 0.0002	347 ± 6	0.18 ± 0.02
SFP-30	86 ± 5	85 ± 4	0.0321 ± 0.0003	490 ± 4	0.18 ± 0.02
PUR foam composites with LG modified SFP filler					
SFP-10/LG	42 ± 3	88 ± 3	0.0336 ± 0.0003	177 ± 7	0.22 ± 0.02
SFP-20/LG	54 ± 4	90 ± 2	0.0328 ± 0.0004	217 ± 6	0.23 ± 0.02
SFP-30/LG	68 ± 6	92 ± 2	0.0319 ± 0.0002	321 ± 7	0.24 ± 0.02
PUR foam composites with TCPP and SFP filler					
SFP-10/TCPP	56 ± 5	88 ± 3	0.0321 ± 0.0002	334 ± 10	0.15 ± 0.03
SFP-20/TCPP	75 ± 3	89 ± 2	0.0321 ± 0.0002	378 ± 12	0.16 ± 0.02
SFP-30/TCPP	95 ± 5	91 ± 3	0.0295 ± 0.0003	462 ± 8	0.18 ± 0.03
PUR foam composites with TCPP and LG modified SFP filler					
SFP-10/LG/TCPP	51 ± 4	83 ± 2	0.0320 ± 0.0004	306 ± 8	0.17 ± 0.03
SFP-20/LG/TCPP	56 ± 2	90 ± 2	0.0325 ± 0.0003	313 ± 5	0.21 ± 0.03
SFP-30/LG/TCPP	73 ± 3	92 ± 2	0.0291 ± 0.0002	353 ± 9	0.24 ± 0.03

**Table 3:** Thermal degradation parameters of PUR and flame retardant PUR foams.

Foam	T_5wt.%_, °C	T_50wt.%_, °C	T_max_			Char Yield at 600 °C, wt.%
			1st Stage	2nd Stage	3rd Stage	
Control PUR foams						
SFP-0	221	513	219	321	459	35.3
SFP-0/TCPP	207	467	221	327	469	34.5
PUR foams with SFP filler						
SFP-10	217	499	221	315	457	34.0
SFP-20	215	483	219	315	461	32.3
SFP-30	215	481	219	319	455	33.7
PUR foams with LG-modified SFP filler						
SFP-10/LG	217	507	223	313	461	33.6
SFP-20/LG	209	485	217	309	465	30.3
SFP-30/LG	215	501	217	313	463	31.9
PUR foams with TCPP and SFP filler						
SFP-10/TCPP	199	469	215	321	461	33.6
SFP-20/TCPP	191	443	215	319	457	30.4
SFP-30/TCPP	189	425	219	317	451	30.7
PUR foams with TCPP and LG-modified SFP filler						
SFP-10/LG/TCPP	201	473	219	319	465	33.3
SFP-20/LG/TCPP	194	471	213	317	465	32.6
SFP-30/LG/TCPP	197	473	215	317	457	30.1

**Table 4:** Ignitability test results of PUR and flame retardant PUR foams.

Foam	Time for Flame to Reach 150 mm Height, s	Height of Flame Damaged Area, mm	Self-Extinguishment Time after Flame Source Removal, s
SFP-0	6 ± 1	180 ± 2	–
SFP-0/TCPP	Did not reach	30 ± 3	Instantly
SFP-10	6 ± 2	180 ± 2	–
SFP-20	5 ± 2	187 ± 4	–
SFP-30	4 ± 1	190 ± 4	–
SFP-10/LG	Did not reach	46 ± 2	Instantly
SFP-20/LG	Did not reach	44 ± 3	Instantly
SFP-30/LG	Did not reach	29 ± 2	Instantly
SFP-10/TCPP	Did not reach	25 ± 3	Instantly
SFP-20/TCPP	Did not reach	16 ± 3	Instantly
SFP-30/TCPP	Did not reach	25 ± 3	Instantly
SFP-10/LG/TCPP	Did not reach	19 ± 2	Instantly
SFP-20/LG/TCPP	Did not reach	21 ± 2	Instantly
SFP-30/LG/TCPP	Did not reach	23 ± 3	Instantly

**Table 5:** Cone calorimeter test data of PUR and flame retardant PUR foams.

Foam	HRR, kW/m^2^	TSR, m^2^/m^2^	COY, kg/kg	CO_2_Y, kg/kg	COY/CO_2_Y, n.d.	Ignition Time, s	LOI, %
Control PUR foams							
SFP-0	99	1139	0.17	3.65	0.05	4	19.8
SFP-0/TCPP	113	1968	0.36	4.24	0.08	6	20.8
PUR foams with SFP filler							
SFP-10	208	3412	0.30	5.88	0.05	2	19.4
SFP-20	238	3383	0.31	5.62	0.06	2	19.2
SFP-30	205	3796	0.26	6.41	0.04	2	18.9
PUR foams with LG-modified SFP filler							
SFP-10/LG	168	2026	0.19	6.04	0.03	4	20.2
SFP-20/LG	180	2626	0.19	6.12	0.03	4	20.5
SFP-30/LG	198	3291	0.27	6.19	0.04	4	20.6
PUR foams with TCPP and SFP filler							
SFP-10/TCPP	148	2918	0.38	4.18	0.09	6	20.8
SFP-20/TCPP	170	2686	0.42	4.53	0.09	6	21.5
SFP-30/TCPP	134	4232	0.33	4.27	0.08	4	20.1
PUR foams with TCPP and LG-modified SFP filler							
SFP-10/LG/TCPP	168	2795	0.39	4.71	0.08	6	21.7
SFP-20/LG/TCPP	175	3905	0.32	4.58	0.07	6	21.7
SFP-30/LG/TCPP	130	2898	0.29	3.76	0.08	8	21.5

## Data Availability

Not applicable.
